# Spreading of Beta-Amyloid in Organotypic Mouse Brain Slices and Microglial Elimination and Effects on Cholinergic Neurons

**DOI:** 10.3390/biom11030434

**Published:** 2021-03-15

**Authors:** Kurt Moelgg, Faryal Jummun, Christian Humpel

**Affiliations:** Laboratory of Psychiatry and Experimental Alzheimer’s Research, Department Psychiatry I, Medical University of Innsbruck, Anichstr 35, A-6020 Innsbruck, Austria; kurt.moelgg@student.i-med.ac.at (K.M.); faryal.jummun@student.i-med.ac.at (F.J.)

**Keywords:** Alzheimer, beta-amyloid, spreading, organotypic brain slices, microglia

## Abstract

The extracellular deposition of β-amyloid (Aβ) is one of the major characteristics in Alzheimer´s disease (AD). The “spreading hypothesis” suggests that a pathological protein (similar to prions) spreads over the entire brain. The aim of the present study was to use organotypic brain slices of postnatal day 8–10 mice. Using collagen hydrogels, we applied different Aβ peptides onto brain slices and analyzed spreading as well as glial reactions after eight weeks of incubation. Our data showed that from all tested Aβ peptides, human Aβ_42_ had the most potent activity to spread over into adjacent “target” areas. This effect was potentiated when brain slices from transgenic AD mice (APP_SweDI) were cultured. When different brain areas were connected to the “target slice” the spreading activity was more intense, originating from ventral striatum and brain stem. Reactive glial-fibrillary acidic protein (GFAP) astrogliosis increased over time, but Aβ depositions co-localized only with Iba1+ microglia but not with astrocytes. Application of human Aβ_42_ did not cause a degeneration of cholinergic neurons. We concluded that human Aβ_42_ spreads over into other “target areas”, causing activation of glial cells. Most of the spread Aβ_42_ was taken up by microglia, and thus toxic free Aβ could not damage cholinergic neurons.

## 1. Introduction

The life expectancy of humans has markedly increased over the last 100 years. As age is the main risk factor for Alzheimer’s disease (AD), the number of patients suffering from AD and mixed forms of dementia will dramatically increase over the next 50 years. It is expected that there will be about 80 million AD patients worldwide in 2050. AD is characterized by severe β-amyloid (Aβ) deposition in the brain (extracellular plaques), tau pathology (hyperphosphorylated tau causes neurofibrillary tangles (NFTs)), cell death of cholinergic neurons (loss of the neurotransmitter acetylcholine), astroglial and microglial activation, inflammation and cerebrovascular damage. It is well established that Aβ deposits are also found in brain vessels, the so-called cerebral amyloid angiopathy [1]. The causes of AD are yet unknown but at present the Aβ cascade, which is the most prominent hypothesis [2], is surrounded by more and more controversy [3,4]. So far it is not entirely clear when and how the AD pathology starts, but there is growing evidence that spreading of toxic, low molecular weight Aβ oligomers plays a central role [5,6].

### 1.1. Beta-Amyloid Plaques in Alzheimer’s Disease

The amyloid precursor protein (APP) is a large transmembrane protein that is processed into different Aβ peptides by secretases. The 42 amino acid peptide (Aβ_42_) especially is toxic and can aggregate and form the typical plaques in the AD brain. The plaques consist of a central core of highly aggregated Aβ peptides and a halo surrounding the plaques [7,8], which contains degenerating nerve fibers and several infiltrating cells, such as reactive astrocytes or microglia. It is well established that brain vessels are also associated with plaques [7,8], but their role remains unclear. In AD, aberrant Aβ production might trigger tau processing and sorting, to the extent that tau is found in hyperphosphorylated and aggregated forms [9,10,11,12,13]. At present it is not known when and how the pathological cascade is initiated. It has been suggested that the Aβ pathology is transmitted similarly to how prions act [5,14,15,16]. In fact, Meyer-Luehmann et al. [17,18] have shown that brain extracts from human AD patients or AD mice can induce the AD plaque pathology. There is clear evidence that initial transformation of a critical concentration of monomers to small soluble oligomers is a crucial step in generating the neurotoxic intermediate that drives the pathogenesis [6].

### 1.2. Spreading in Organotypic Brain Slices

AD is usually studied in vivo with transgenic animals overexpressing APP. Many such animals have been developed, and we have also gathered good experience using a mouse model of such a type, in which APP with a Swedish-Dutch-Iowa mutation (APP_SweDI) is overexpressed [7,19,20]. However, these mice have limitations. First, AD is >99% a sporadic disease and not a genetic disease; thus, these mice only partially reflect sporadic AD pathologies. Second, these mice mostly only reflect single pathologies; and third, sporadic models for AD have not been described so far [21]. We are highly interested in establishing and characterizing AD pathologies ex vivo in order to study progression of AD pathologies, as well as the stimuli that induce the pathology and factors that protect or counteract the pathologies. In our research group, we have been using organotypic brain slices for almost 25 years now. Organotypic brain slices are three-dimensional, thin sections (150–400 µm) from a brain and are cultured for several weeks [22,23,24]. Cultivation of organotypic brain slices from AD transgenic mice is a novel and innovative technique that has been successfully used by us and also by others [22,23,24,25,26,27,28]. The major advantage of brain slices is the ability to combine two or more brain areas and study them in an isolated system over several weeks. Such ex vivo brain slice models are extremely efficient for the study of spreading of AD-like pathologies from one brain area to another.

In this study, we prepared half-brain slice cultures from postnatal mice (DONOR) and combined them in direct connection with postnatal slices from another area (TARGET). Afterwards, we applied Aβ peptides as a bolus, using well-characterized collagen hydrogels to guarantee local, focused, stable and slow release of the peptides. We show that the toxic human Aβ_42_ peptide can indeed spread to other brain areas. Additionally, we show that spreading is potentiated in AD transgenic mice and that Aβ is eliminated by microglia, thereby protecting cholinergic neurons.

## 2. Materials and Methods

### 2.1. Organotypic Brain Slices

In this study, wildtype (C57BL/6N) and transgenic APP_SweDI (expressing the amyloid precursor protein harboring the Swedish K670N/M671L, Dutch E693Q and Iowa D694N mutations; C57BL/6-Tg (Thy1-APPSwDutIowa) BWevn/Mmjax) mice were used. They were housed in the animal facility at the Medical University of Innsbruck with open access to food and water and 12 h/12 h light–dark cycles were used. Transgenic mice produce Aβ plaques at an age > 6 months [29] and we have extensive experience with transgenic AD mouse models [19]. All animal experiments were approved by the Austrian Ministry of Science and Research (66.011/0055-WF/V/3b/2017) and conformed to the Austrian guidelines on animal welfare and experimentation. Our study using animals (mice) followed ethical guidelines for killing animals and our animal work was in compliance with international and national regulations. All work was performed according to the principles of the Three Rs (reduce, refine, replace) for animal experiments. All our slice experiments were defined as “organ removal” and were not “animal experiments”.

**Figure 1:**
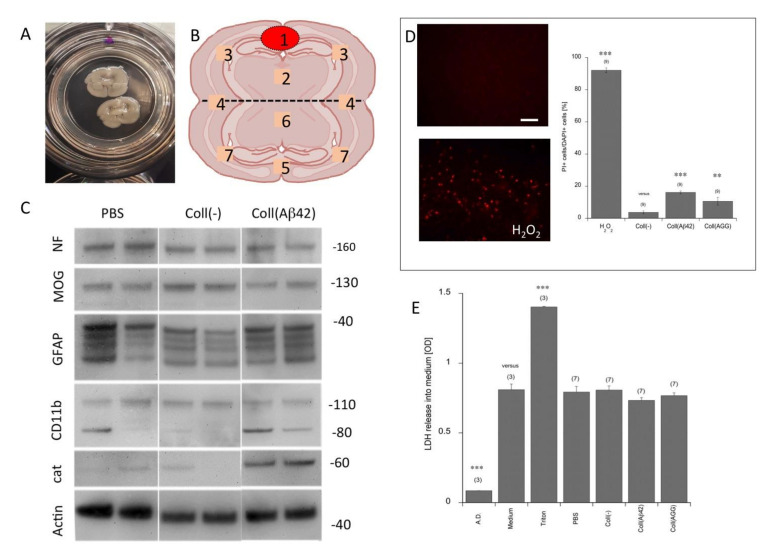
*Scheme of the organotypic brains slices and viability.* Coronal organotypic brain slices were prepared from postnatal day 8–10 mice and placed on 0.4 µm semipermeable membrane inserts (**A**). (**B**) The schematic connection of an upper “donor slice” and a lower “target slice”. The red circle shows placement of the collagen hydrogel loaded with beta-amyloid peptides. The values 1–7 give the areas which were analyzed. Western blot analysis (from two independent mice) showed a stable expression of neurofilament (NF), myelin oligodendrocyte protein (MOG) and glial fibrillary acidic protein (GFAP) (**C**). Microglial CD11b was weakly expressed in all slices, with a somewhat higher density in slices where the beta-amyloid was applied, but showed a high heterogeneity between slices (**C**). Catalase was very weak, but markedly increased in slices where beta-amyloid was applied (**C**). Actin served as a loading control. Propidiumiodide (PI) staining showed a weak background in all slices, and no marked changes between the groups. As a positive control for the assay, slices were incubated with H_2_O_2_ for three days (**D**). Lactate dehydrogenase (LDH) release into medium showed no marked changes between the groups, while the positive control (2% Triton) markedly increased LDH release into medium after one day (**E**). Values are given as means ± SEM; values in parenthesis give the number of analyzed animals. Statistical analysis was performed using ANOVA with a Fisher LSD post hoc test, where *p* < 0.05 represents significance versus a control (** *p* < 0.01, *** *p* < 0.001; n.s., not significant). Scale bar in D = 450 µm (**A**), 60 µm (**D**). PBS, phosphate buffered saline; Coll(–), empty collagen hydrogel, Aβ, beta-amyloid; AGG, aggregated, A.D., aqua destillatum.

Organotypic brain slices were prepared as described previously by us in detail [22,23,30]. Briefly, postnatal day 8–10 mice were rapidly sacrificed and their brains dissected and glued (Glue Loctite 404 or 401) onto a chuck of a water-cooled vibratome Leica VT1000A, which was then triggered close to a commercial razor blade. Under aseptic conditions, 150 µm thick coronal sections were cut at the hippocampal level into halves and collected in sterile medium. The organotypic slices were carefully placed onto a 0.4 µm membrane insert (Millipore PICM03050, Darmstadt, Germany) within a 6-well plate (Figure 1A). Slices were cultured in 6-well plates (Greiner, Kremsmünster, Austria) with 1.2 mL/well of the following culture medium: 50% MEM/HEPES (Gibco, Vienna, Austria), 25% heat inactivated horse serum (Gibco/Lifetech, Vienna, Austria), 25% Hanks’ solution (Gibco), 2 mM NaHCO_3_ (Merck, Austria), 6.5 mg/mL glucose (Merck, Darmstadt, Germany), 2 mM glutamine (Merck, Darmstadt, Germany), pH 7.2. Co-slices were prepared as shown in Figure 1B and incubated at 37 °C and 5% CO_2_, changing the medium once a week. After one-week collagen hydrogels were applied to the slice and then cultured for a further eight weeks, the brain slices were fixed in 4% paraformaldehyde for 3 h and stored at 4 °C in PBS until used.

The viability of the slices was tested using (a) propidiumiode (PI) staining, by incubating living slices with 2 µg/mL PI in medium for 30 min, washing them and then photographing them under the red fluorescence filter as described by us previously [31]; (b) release of lactate dehydrogenase (Roche 1644793) into the medium and measurement of activity by incubating 100 µL medium with 100 µL working solution and measuring optical density at 450 nm, as described by us previously [31]; and (c) Western blot of neuronal and glial viability markers.

In order to study the spreading, a “donor (half-brain) slice” was always connected to a “target (half-brain) slice” taken from wildtype C57BL6 mice (Figure 1B). In additional experiments, slices from transgenic (APP_SweDI) mice were also prepared (Figure 6A–D). For further experiments, slices from the dorsal striatum and ventral (limbic) region and brain stem were also connected as a “donor slice” (Figure 7A–D). With coronal brain stem slices being difficult to prepare, the brain stem was dissected, 400 µm thick chopper slices were prepared and two to three slices (as donor slices) were connected to the hippocampal target slice. In an additional set of experiments, cholinergic neurons of the nucleus basalis of Meynert (nBM) were also prepared, as described in detail previously [32].

### 2.2. Preparation of Collagen Hydrogels

In order to apply the substances onto the donor slices, non-toxic, degradable and slow-releasing collagen hydrogels were placed on the slices. The collagen hydrogels were prepared using 4S-Star-Poly(ethylene Glycol) Succinimidyl Succinate (Merck-Sigma JKA 7006; 4S-StarPEG, Darmstadt, Germany) as a cross-linker, following our detailed description [32]. Briefly, 0.4 mg bovine collagen type I (Collagen Solutions^®^, Glasgow, UK) was linked with 0.8 mg 4S-StarPEG in phosphate buffered saline (PBS) at pH 7.4, obtaining a final hydrogel of 2 mg/mL collagen and 1:2 molar ratio of coll:4S-StarPEG. Drops of 2 µL were pipetted onto Teflon tapes and gelled for 1 h at 37 °C. To visualize the collagen hydrogels on the brain slices, 10 µL of an anti-rabbit Alexa-488 antibody (Invitrogen, Vienna, Austria) was loaded before cross-linking. When loading Aβ peptides, 10 µL of a peptide stock (1 mg/mL) was added to the collagen hydrogels before crosslinking, giving 100 ng/2 µL hydrogel. For aggregated Aβ, only 10 ng/2 µL collagen hydrogel spot was added due to methodological limits.

In the present study we used different Aβ peptides: human Aβ_40_ (Innovagen SP-BA40-1, Lund, Sweden), human Aβ_42_ (Innovagen AP-BA-42-1), pyr3Glu-Aβ (Innovagen SP-5046-5), Aβ_(25-35)_ fragment (Sigma A4559) and reversed human Aβ_(42-1)_ (Innovagen SP-BA42R-1). The aggregation of human Aβ_42_ was performed as described by us [33,34]. Briefly, human Aβ_42_ was dissolved in 1 mg/mL Tris-HCl pH 9.0, diluted 1+1 with PBS+ 0.05% sodiumdodecylsulfate, giving a concentration of 100 µM, and incubated overnight at 4 °C. It was then diluted again 1:10 with PBS and incubated for two weeks at 4 °C, yielding a final concentration of 50 µg/mL (10 µM).

### 2.3. Immunohistochemistry

Immunohistochemistry was performed under free-floating conditions as previously described [35]. The fixed slices were washed with PBS and incubated in PBS/0.1% Triton (T-PBS) for 30 min at 20 °C while shaking. To quench endogenous peroxidase, slices were treated with PBS/1% H_2_O_2_/5% methanol. After the incubation, the slices were blocked in T-PBS/20% horse serum (GIBCO Invitrogen, Vienna, Austria)/0.2% BSA (SERVA, Vienna, Austria) for 30 min at 20 °C while shaking. Following the blocking, brain sections were incubated with primary antibodies (see below) in T-PBS/0.2% BSA for two days at 4 °C. The slices were then washed and incubated with the corresponding biotinylated antibodies (1:200, Vector Laboratories, Invitrogen, Vienna, Austria) in T-PBS/0.2% BSA for 1 h at 20 °C while shaking. After the secondary antibody incubation, the slices were rinsed with PBS and incubated in avidin-biotin complex solution (Elite ABC kit, Vector Laboratories) for 1 h at 20 °C while shaking. Finally, the slices were washed with 50 mM Tris-buffered saline (TBS) and then incubated in 0.5 mg/mL 3,3’-diaminobenzidine (DAB, Sigma)/TBS/0.003% H_2_O_2_ at 20 °C in the dark until a signal was detected. Once DAB staining was visible, the reaction was stopped by adding TBS to the slices. The brain slices were rinsed with TBS, inserted into a 6-well plate, covered with Vectashield (Vector), coverslipped and subsequently analyzed with an inverted light microscope (Leica DM IRB, Vienna, Austria). Some slices were fluorescently stained with Alexa-488 (green fluorescent) or Alexa-546 (red fluorescent) antibodies and counterstained with the blue fluorescent nuclear dye DAPI.

In order to detect Aβ-like immunoreactivity, we used the Aβ antibody clone 6E10 (BioLegend, Vienna, Austria, 803015, 1:1000, secondary antibody is mouse), which recognizes the epitope 1-16 of human Aβ. This antibody does not recognize mouse Aβ because 3 amino acids (positions 5-10-13) differ between murine and human Aβ. An antibody against glial-fibrillary acidic protein (GFAP) was used to detect astrocytes (Millipore AB5541, 1:2000, secondary antibody was chicken). For the detection of microglia, the Iba1 antibody was used (Wako 019-19741; Fujifilm, Neuss, Germany, 1:500; secondary antibody is rabbit). An antibody against choline acetyltransferase (ChAT, Millipore AB144P, 1:750, secondary antibody was goat) was used to label cholinergic neurons.

### 2.4. RNA Isolation and Quantitative TaqMan-PCR

Quantitative RT-PCR was performed as described by us [36]. The brain slices were scraped from the membrane and a pool of two was transferred to 600 µL of buffer RLT (RNeasy Mini Kit; Qiagen, Vienna Austria) and homogenized using an ultrasonic device (Hielscher Ultrasonic Processor, Teltow, Germany), before being further disrupted using QIAshredder columns (Qiagen). All RNA was extracted with the RNeasy Mini Kit (Qiagen). RNA concentrations were determined photometrically using BioPhotometer 6131 (Eppendorf). Reverse transcription was performed on 250 ng of total RNA using the Omniscript Reverse Transciptase Kit (Qiagen), random hexamer primers (Promega, Vienna, Austria) and RNAse Inhibitor (Sigma). The reverse transcription mix was incubated for 60 min at 37 °C. The relative expression of mouse *app* (Applied Biosystems, Thermo Scientific, Vienna Austria, Mm01344172_m1) was obtained by TaqMan quantitative PCR (qRT-PCR) using a standard curve method based on PCR products of known concentration correlated to the house keeping gene *Glycerinaldehyd-3-phosphat-dehydrogenase* (*GAPDH,* Mm99999915_g1, Applied Biosystems). qRT-PCR (50 cycles) was performed in duplicates using 1 µL total RNA equivalents of cDNA and the specific TaqMan gene expression assay for each 20 μL reaction in TaqMan Universal PCR Master Mix (Applied Biosystems). Analysis was performed with QuantStudio 6 (Applied Biosystems). The Ct values for each gene expression assay were recorded for each individual preparation. Eventually, normalized molecule numbers were calculated for each gene from their respective standard curve.

### 2.5. Western Blots

After incubation, two brain slices were collected in an Eppendorf tube and homogenized in 100 µL PBS with a protease inhibitor cocktail (P-8340, Sigma) by sonication. The solution was centrifuged at 14,000× *g* for 6 min at 4 °C and the supernatant was collected. The total protein amount in the samples was determined using the Bradford assay with Coomassie brilliant blue G250 dye (Bio-Rad, Vienna Austria). Twenty µL (equals 20 µg protein per lane) were directly loaded (native) onto 10% Bis-Tris polyacrylamide gel (Invitrogen) (Aβ, myelin oligodendrocyte protein (MOG), microglial CD11b, catalase) or denatured (10 min, 70 °C, with reducing agent) and then loaded (GFAP, neurofilament, actin). Electrophoresis was performed for 35 min at 200 V. Samples were electrotransferred onto PVDF membranes for 20 min at 25 V in a semi-dry transfer cell (Thermo Scientific, Vienna, Austria). Blotting was done by using a WesternBreeze Chemiluminescent immunodetection system (Invitrogen). Blots were blocked with blocking buffer for 30 min and incubated overnight on a shaker at 4 °C with primary antibodies against neurofilament (1:10,000, Novus NB300-135), MOG (1:2,000, proteintech 12690-1-AP), CD11b (1:2,000, proteintech 20991-1-AP, TPH, Vienna Austria), catalase (1:10,000, Thermo PA1-28372), Aβ (1:5,000), GFAP (1:2,000, Millipore AB5541) and actin (1:1,000, Sigma A2066) as a loading control. Subsequently, blots were briefly washed and incubated with alkaline phosphatase-conjugated secondary antibody (mouse for Aβ, chicken for GFAP and rabbit for all others) for 30 min at room temperature. Afterwards, the blots were briefly washed again, incubated for 15 min in CDP-Star chemiluminescent substrate solution (Roche) and visualized with a cooled CCD camera (SearchLight, Thermo Scientific).

### 2.6. Release Experiments

In order to measure the time-dependent release of Aβ from collagen hydrogels, 10 µg of the peptide was loaded in 200 µL collagen hydrogel solution as described above; 2×2 µL drops of this collagen hydrogel solution (200 ng in total) were then placed onto a small piece of Parafilm, which was thereafter placed in a 24-well plate in 500 µL brain slice medium. This plate was incubated at 37 °C for up to eight weeks and at each time point the medium was frozen at –20 °C until use. The release of Aβ_40/42_ in the brain slice medium was measured using automated Lumipulse technology (Fujirebio G600II, Hannover, Germany).

### 2.7. Data Analysis and Statistics

*Evaluation of plaques:* Brain sections at the cortical level were analyzed as reported by a blinded investigator [24]. The brain slices were photographed with the Leica inverse microscope at a 10x magnification under a red filter. The exposure time was always 23 ms with the bright light set at the lowest level. The software OpenLab (Improvision, Tübingen, Germany) was used on a Mac computer connected to the microscope. Pictures were saved as JPG files and the analysis was performed using ImageJ. The pictures were transformed to 8-bit grayscale images. The calibration was set at 0.470 (distance in pixels), 1.00 (known distance), 1.0 (pixel aspect ratio) and µm (unit in length) and the global setting was activated. The picture was transformed into a binary image and the threshold was adapted to 30–40. The number of particles was counted, setting the size to 100–8000 pixels. In some sections small (100–400 pixels), medium (400–1000 pixels) and large (1000–8000 pixels) plaques were measured. The number of plaques was counted in a defined circle with an area of 2.5 mm^2^.

*Microscopic quantification of cholinergic neurons:* The number of ChAT+ nBM neurons was counted in six subsequent slices on both sides between Bregma –0.34 and –1.34 as described in detail by us [32]. Neurons were counted when they contained a clear brown (DAB) cytoplasmic staining with at least one neuronal fiber extension. Weakly stained neurons were counted when a nucleus was visible. Round cells without any fiber extensions were excluded.

Statistical analysis was performed by one-way ANOVA with a subsequent Fisher LSD post hoc test, where *p* < 0.05 represented significance.

## 3. Results

### 3.1. Culturing of Brain Slices and Viability

Organotypic brain slices were cultured on semipermeable membranes where they adhered and flattened on the membrane within one week (Figure 1A). In the majority of the experiments, two coronal half-brain slices (a donor and a target slice) were prepared and cultured for eight weeks (Figure 1B). The collagen hydrogel containing the peptides was placed on top of the donor slice (Figure 1B). In order to show the viability of the brain slices after eight weeks of culturing, Western blot, propidiumiodide and LDH analyses were performed. Western blot analysis showed a stable expression of NF, MOG and GFAP (Figure 1C). GFAP displayed a more fragmented pattern, while usually in fresh tissue a single band is seen (Figure 1C). Microglial CD11b was weakly expressed in all slices, with a somewhat higher density in slices where the aggregated Aβ was applied (Figure 1C). Catalase was very weak, but markedly increased in slices where aggregated Aβ was applied (Figure 1C). In general, the majority of the slices showed a homogenous pattern, while only about 10% of slices were damaged or did not flatten and about 10% of the slices showed a different pattern. The damaged, non-flattened and different pattern-showing slices were excluded from this study. PI staining showed a weak background in all slices, and no marked changes between the groups, while the positive control (H_2_O_2_) markedly increased after three days (Figure 1D). LDH release into the medium showed no marked changes between the groups, while the positive control (2% Triton) markedly increased LDH release into the medium after one day (Figure 1E).

### 3.2. Loading and Release of Beta-Amyloid from Collagen Hydrogels

**Figure 2:**
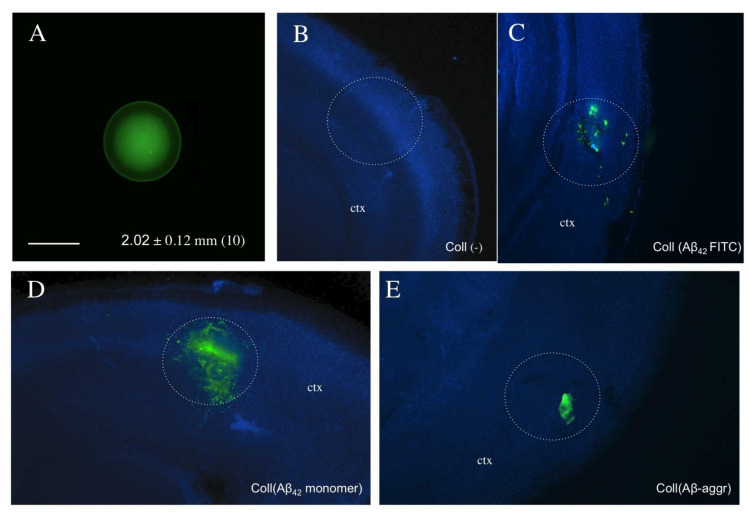
*Characterization of the collagen hydrogel* and placement on a brain slice (white circles). The collagen hydrogel had a size of approximately 2 mm (*n* = 10) and became visible with fluorescently stained green Alexa-488 (**A**). Collagen hydrogels were loaded without (**B**) or with Fluoresceinisothiocyanat (FITC)-labeled beta-amyloid-42 (**C**), or with human beta-amyloid-42 (**D**) or aggregated beta-amyloid-42 (**E**) and placed on brain slices, incubated for two days and then fixed and counterstained with nuclear DAPI (blue). The hAβ42 may exist in monomeric or oligomeric forms or may partly aggregate in the hydrogel (see also Western blots in Figure 4). Only background staining was visible when the empty hydrogel (Coll(–)) was loaded (**B**) and green fluorescence was seen with the FITC-labeled beta-amyloid-42 (**C**). Collagen hydrogel loaded with unconjugated beta-amyloid was made visible by immunostaining using antibodies with anti-mouse A488 (green, **D**,**E**). Scale bar in A = 1.5 mm (**A**–**E**). ctx, cortex.

**Figure 3:**
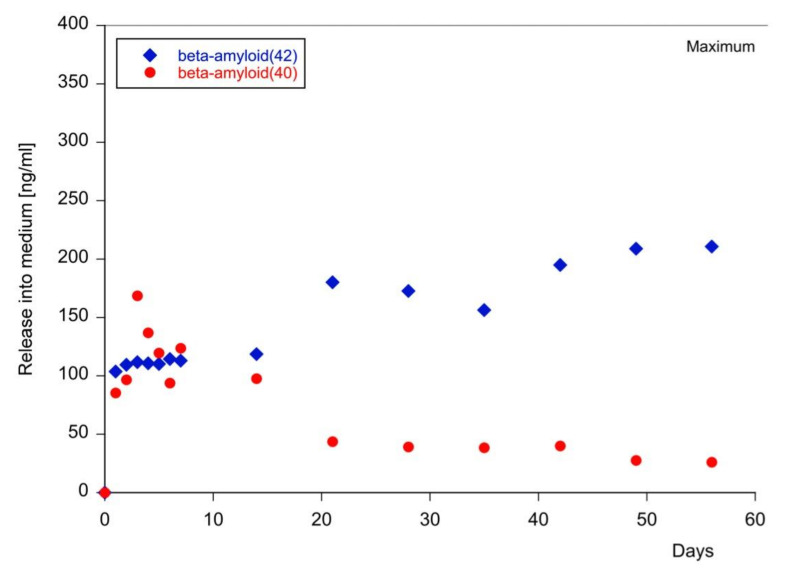
Release of beta-amyloid from collagen hydrogels. In order to measure time-dependent release of beta-amyloid from collagen hydrogels, beta-amyloid-42 (kite) and beta-amyloid-40 (circle) were loaded in collagen hydrogels and placed onto a small piece of Parafilm in medium. The release was measured for up to eight weeks using automated Lumipulse technology. Values are given as ng/mL. The maximal release into the medium was 400 ng/mL (2 × 2 µL hydrogels, 200 ng in 500 µL medium). The experiment was a time-course undertaken once (*n* = 1) but analyzed in duplicate.

Collagen hydrogels are spherical structures with a size of approximately 2 mm that can be easily handled (Figure 2A). Two days after application of collagen hydrogels loaded with fluorescent Aβ (Figure 2C), monomeric Aβ_42_ (Figure 2D) or aggregated Aβ (Figure 2E), strong immunoreactivity was found at the site of location, while only background was seen when an empty collagen hydrogel was placed (Figure 2B). In order to measure the release of Aβ into the medium, collagen hydrogels were loaded with Aβ_40_ or Aβ_42_ on a piece of Parafilm placed into the brain slice medium and the release was measured for up to eight weeks. Aβ_40_ and Aβ_42_ were rapidly released into medium within one day (Figure 3). The release of Aβ_42_ into the medium increased only slightly, to approximately half of the maximal concentration, within eight weeks (Figure 3), whereas the Aβ_40_ levels in the medium decreased over eight weeks (Figure 3).

### 3.3. Western Blot of Monomer and Aggregated Aβ

**Figure 4:**
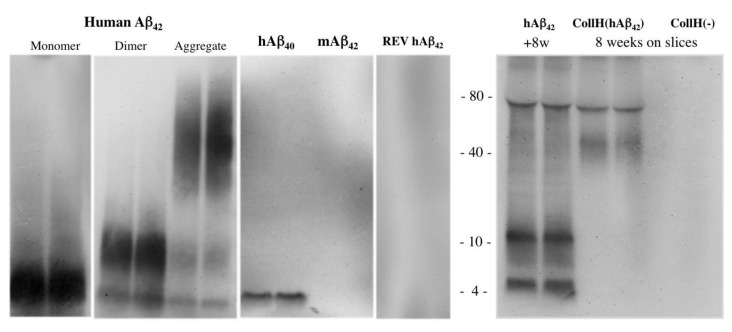
Western blot of beta-amyloid (Aβ). Beta-amyloid peptides (500 ng) were loaded onto gels and stained with a beta-amyloid antibody. Note that the antibody detected a 4 kDa monomer and an 8 kDa dimer peptide of human Aβ_42_ and a 4 kDa peptide of human Aβ_40_ but not mouse Aβ_42_. Human Aβ_42_ was allowed to aggregate and showed a smear of higher 40–80 kDa aggregated species. As a negative control, a reversed (REV) peptide was loaded. When Aβ_42_ was incubated for eight weeks (without slices), three major bands were visible: at 4, 12 and approximately 80 kDa. Slices incubated for eight weeks with a collagen-loaded Aβ_42_ (CollH(Aβ_42_)) hydrogel showed two major bands at approximately 40 and 80 kDa, pointing to aggregation stages during long-term culturing, while no staining was visible in slices incubated with an empty collagen hydrogel (CollH(-)).

The Western blot analysis clearly verified that the 6E10 Aβ antibody recognized human Aβ_42_ and human Aβ_40_, but not mouse Aβ (Figure 4). Western blot also showed spontaneous dimerization of Aβ_42_ but not Aβ_40_ (Figure 4). Aggregation of human Aβ_42_ was also successful and showed several high-order aggregates (Figure 4). No staining was visible when reversed Aβ was loaded (Figure 4). When Aβ_42_ was incubated for eight weeks (without slices), then three major bands were visible: at 4, 12 and approximately 80 kDa (Figure 4). Slices incubated for eight weeks with a collagen-loaded Aβ_42_ (CollH(Aβ_42_)) hydrogel showed two major bands at approximately 40 and 80 kDa, pointing to aggregation stages during long-term culturing, while no staining was visible in slices incubated with an empty collagen hydrogel (CollH(-)) (Figure 4).

### 3.4. Beta-Amyloid Spreading in Wildtype Brain Slices

**Figure 5:**
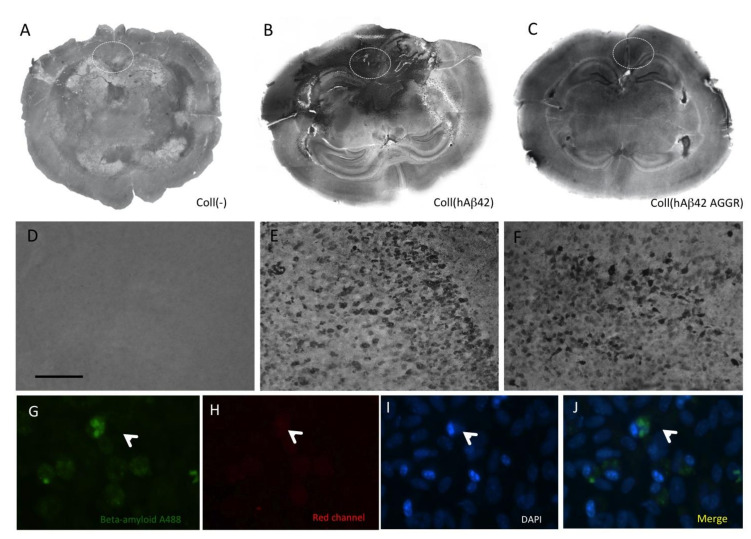
Overview of beta-amyloid (Aβ) spreading in brain slices. Two half-brain slices were connected and after one week the collagen hydrogel (Coll) was loaded without (**A**,**D**) or with (**B**,**E**) human Aβ_42_, or with aggregated (AGGR) Aβ_42_ (**C**,**F**), placed onto the upper “donor slice” and incubated for eight weeks, then fixed and stained with the Aβ antibody. Note the severe Aβ-like immunoreactivity all over the donor slice and also partly in the target slice after application of human Aβ_42_. Severe intracellular Aβ-like immunoreactivity was visible (**E**,**F**). Co-staining of A488-labeled Aβ (green, **G**) with nuclear DAPI (blue, I) showed cellular staining in the cytoplasm (J), while the staining was negative in the red channel (**G**). Scale bar in D = 220 µm (**A**–**C**), 150 µm (**D**–**F**) and 50 µm (**G**–**J**).

In order to demonstrate spreading of Aβ in brain slices, the peptides were placed in a collagen hydrogel on top of a donor slice and the spreading was observed in both the donor and the target slice. The application of PBS as a control or only an empty collagen hydrogel showed only background staining (Figure 5A,D, Table 1). From all tested peptides, only human Aβ_42_ showed strong spreading into other brain areas (Figure 5B,E, Table 1). Quantification was performed in area 1 (location of the hydrogel), in areas 2 and 3 (donor slice), area 4 (borders) and areas 5–7 (target slice). The immunoreactivity was strongest in the area of the placement of the collagen (area 1), markedly increased in adjacent area 2 and in the adjacent cortex (area 3) and was also detectable at the border of the two slices (area 4) and in the target slice (areas 5, 6 and 7) (see Table 1). The application of aggregated human Aβ_42_ showed a similar pattern (Figure 5C,F, Table 1). Application of human Aβ_42_ alone without any collagen hydrogel showed only a very weak staining in adjacent area 2, which was also observed with pyr-Glu-Aβ (Table 1). No immunostaining was seen after application of human Aβ_40_, Aβ_(25-35)_ and mouse Aβ_42_ (Table 1). The application of reversed Aβ showed some immunoreactive staining in distinct areas (Table 1). Co-staining of Aβ (Alexa-488, green) with nuclear DAPI (blue) clearly showed that the Aβ_42_ immunoreactivity was cellular and taken up by cells (Figure 5G–J). The staining was also specific as it was only seen in the green and not in the red channel (Figure 5H).

### 3.5. Beta-Amyloid Spreading in Transgenic APP_SweDI Brain Slices

**Figure 6:**
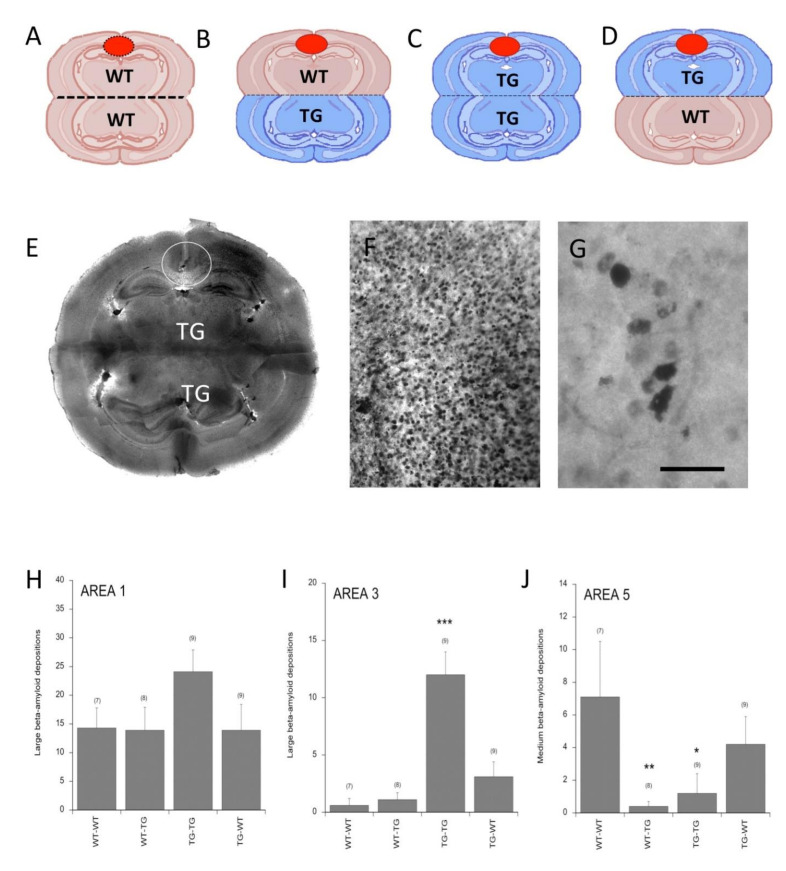
Beta-amyloid spreading in slices taken from transgenic (TG) mice (APP_SweDI). Coronal half-brain co-slices were prepared from wildtype (WT) (donor)–wildtype (**A**), wildtype (donor)–transgenic (**B**), transgenic (donor)–transgenic (**C**) and transgenic (donor)–wildtype (**D**) mice, and after one week collagen hydrogels loaded with human beta-amyloid-42 were applied and slices incubated for eight weeks. An overview showed strong beta-amyloid staining all over the two brain slices taken from transgenic mice (**E**). The hydrogel is marked with a white circle (**E**). A higher magnification is given in (**F**) and (**G**) from the cortical area. The number of beta-amyloid depositions was evaluated in area 1 (**H**), area 3 (**I**) and area 5 (**J**). Computer-assisted analysis of large (**H**,**I**) and medium (**J**) depositions was performed and values are given as means ± SEM; values in parenthesis give the number of animals. Statistical analysis was performed by one-way ANOVA with a subsequent Fisher LSD post hoc test compared to the WT–WT slices (* *p* < 0.05; ** *p* < 0.01; *** *p* < 0.001). Scale bar in G = 250 µm (**E**), 480 µm (**F**) and 80 µm (**G**).

In the next step, we wanted to investigate whether the spreading pattern differed when we combined brain slices taken from transgenic (TG) AD mice and wildtype (WT) mice with each other. Thus, we connected wildtype–wildtype (Figure 6A), wildtype–transgenic (Figure 6B), transgenic–transgenic (Figure 6C) and transgenic–wildtype mouse brain slices (Figure 6D), placed a collagen hydrogel with human Aβ_42_ on the donor slice and analyzed spreading after eight weeks. Application of Aβ onto slices from transgenic mice markedly displayed a strong Aβ-like immunoreactivity all over the brain slices (Figure 6E,F), clearly showing cellular depositions (Figure 6G). Quantitative analysis revealed no differences in large depositions in area 1 (where the hydrogel was placed) in all groups (Figure 6H). The number of large depositions was significantly higher in area 3 when Aβ was applied to slices from transgenic mice paired to transgenic mice (TG-TG group) (Figure 6I). In contrast, the number of medium-sized depositions decreased in area 5 in all groups (tendency in the TG-WT group) (Figure 6J).

### 3.6. Beta-Amyloid Spreading in Different Brain Areas

**Figure 7:**
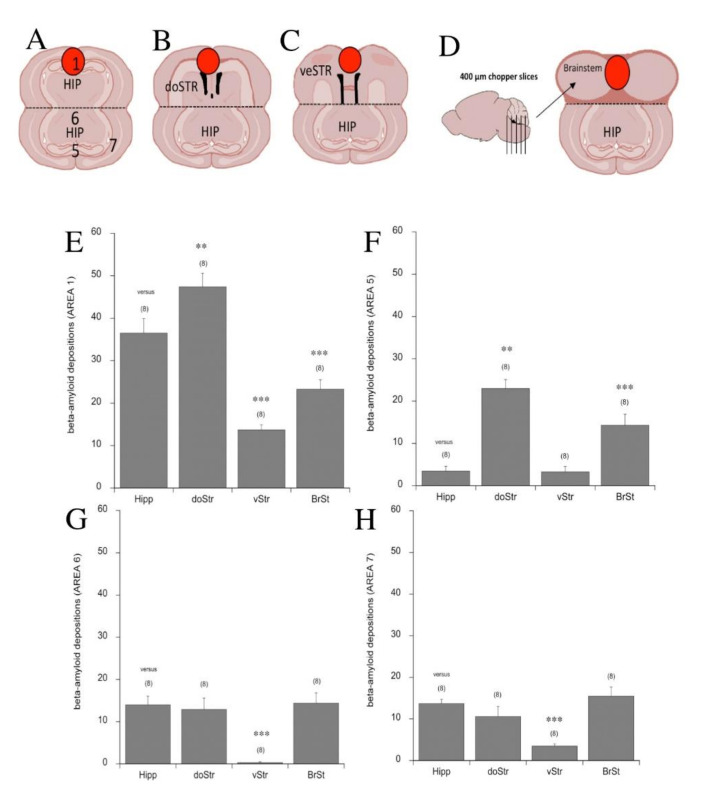
*Beta-amyloid spreading in different brain areas*. The hippocampal (HIP) target brain slice was connected to the hippocampal (HIP) slice (**A**), to the dorsal striatum (doSTR) slice (**B**), the ventral striatal (veSTR) slice containing limbic areas and the amygdala and piriform cortex (**C**) and the brainstem (**D**). As it was difficult to prepare coronal slices of brain stem, we prepared 400 µm-thick chopper slices from brain stem and connected two to three such slices to the target hippocampal slice (**D**). Quantifications of beta-amyloid depositions were performed in area 1 (**A**,**E**), area 5 (**A**,**F**), area 6 (**A**,**G**) and area 7 (**A**,**H**). Values are given as means ± SEM; values in parenthesis give the number of animals. Statistical analysis was performed by one-way ANOVA with a subsequent Fisher LSD post hoc test compared to the hippocampal target slices (** *p* < 0.01; *** *p* < 0.001).

In a next step, we wanted to investigate whether the spreading pattern of human Aβ_42_ was dependent on the target brain areas and we therefore connected the coronal hippocampal slice with another hippocampal slice (Figure 7A), a slice containing the dorsal striatum (Figure 7B), a slice containing the ventral limbic areas (Figure 7C) or chopper slices from the brain stem (Figure 7D). Placement of the Aβ-loaded collagen hydrogel onto the dorsal striatum increased the deposition only in area 1 (hydrogel location) and in area 5 (target hippocampus) (Figure 7E,F). Application of the Aβ collagen-loaded hydrogel onto the ventral (limbic) striatum increased deposition in area 1 (hydrogel location) and significantly decreased the deposition in areas 6 and 7 of the target slice (Figure 7E,G,H). The placement of the Aβ collagen-loaded hydrogel onto the brain stem decreased the deposition in area 1 (hydrogel location) and increased it in area 5 (target hippocampus) only (Figure 7E,F).

### 3.7. qRT-PCR of Endogenous APP

**Figure 8:**
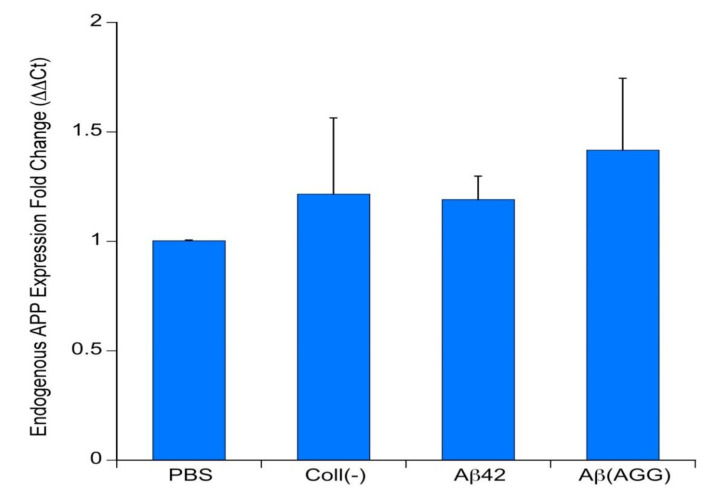
*qRT-pPCR of endogenous amyloid precursor protein (APP)*. Co-slices of wildtype mice were prepared and loaded with phosphate buffer saline (PBS) only, an empty collagen hydrogel (Coll(-)), a collagen hydrogel loaded with human beta-amyloid-42 (Aβ42) or an aggregated beta-amyloid-42 (Aβ(AGG)). After eight weeks in incubation, slices were taken, RNA isolated and a qRT-PCR was done for endogenous mouse *app* gene and compared to the house keeping gene *gapdh*. Note that the treatment did not affect endogenous APP expression as evaluated by one-way ANOVA and Fisher LSD post hoc test.

In order to demonstrate if the application of human Aβ_42_ can induce endogenous mouse APP expression, qRT-PCR was performed. The data showed that neither an empty collagen hydrogel nor application of monomeric or aggregated Aβ_42_ induced endogenous mouse APP expression in comparison to a PBS control (Figure 8).

### 3.8. Astroglial Responses

**Figure 9:**
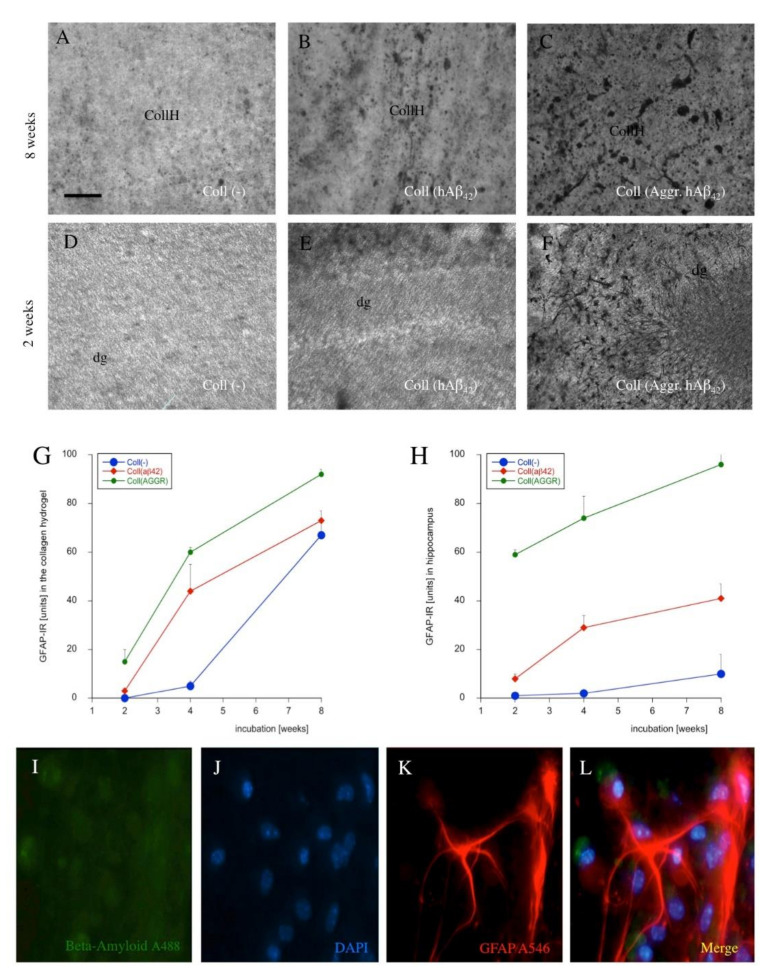
*Astroglial responses stained for glial fibrillary acid protein (GFAP).* Co-slices were loaded with an empty collagen hydrogel (Coll(-)), **A**,**D**), human beta-amyloid(42) (Coll(hAβ_42_); **B**,**E**) or aggregated beta-amyloid (Coll(Aggr.hAβ_42_); **C**,**F**) and analyzed in the area where the hydrogel was placed (area 1) after eight weeks (**A**–**C**) or in the hippocampal area (area 2) after two weeks (**D**–**F**). Quantification over time is given in (**G**) (in area 1) and (**H**) (hippocampal area 2). Values are given as mean ± SEM (*n* = 3). Co-staining of beta-amyloid (**I**, A488, green) and GFAP (**K**, A546, red) with nuclear DAPI (**J**, blue) showed that the beta-amyloid-like immunostaining did not co-localize with GFAP+ astrocytes (**L**, merge). Scale bar in A = 90 µm (**A**–**C**,**D**–**F**) and 40 µm (**I**–**L**).

To examine whether astroglia are involved in the spreading process, GFAP staining was performed. Very weak GFAP staining was seen after application of an empty collagen hydrogel in area 1 after eight weeks (Figure 9A) and after two weeks (Figure 9D). GFAP immunoreactivity slightly increased in the hippocampus after application of monomeric human Aβ_42_ at eight weeks (Figure 9B) and at two weeks (Figure 9E). Application of aggregated human Aβ_42_ resulted in a more severe activation of astroglial GFAP in the hippocampus after eight weeks (Figure 9C) and after two weeks (Figure 9F). A time graph clearly showed an increase within the cultivation period of eight weeks, which was more pronounced in the area where the hydrogel was placed (Figure 9G) and more prominent when aggregated Aβ was applied (Figure 9H). Co-staining of Aβ_42_ (Alexa-488, green) with GFAP (Alexa-546, red) and nuclear DAPI (blue) clearly showed that the Aβ deposits were not located in astrocytes (Figure 9I–L). Quantitative analysis did not show a single co-localization of Aβ and GFAP+ astrocytes (6.4 ± 0.6 astrocytes/field counted, *n* = 6).

### 3.9. Microglial Responses

**Figure 10:**
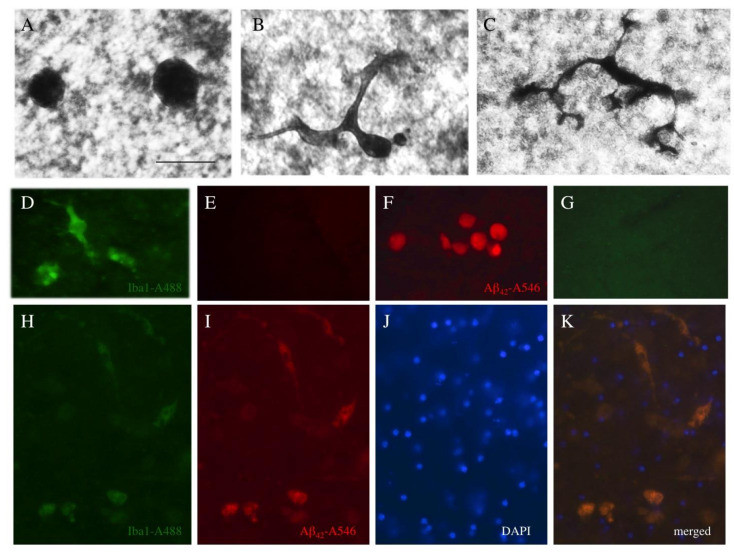
*Microglial* responses. Coronal half-brain slices were prepared and collagen hydrogels were loaded with human beta-amyloid-42 (Aβ42), placed on slices taken from wildtype mice, cultured for eight weeks, fixed and stained for microglial Iba1+. Round (**A**) and ramified (**B**,**C**) Iba1+ microglia could be identified. Immunostaining for Iba1+ Alexa488 (green channel) was selective, as it was not seen in the red channel (**D**,**E**), and also staining for beta-amyloid-Alexa546 (red channel) was specific, as it was not seen in the green channel (**F**,**G**). Co-localization experiments with Iba1 (A488, green, **H**), Aβ (A546, red, **I**) and nuclear DAPI (blue, **J**) showed that the majority of Iba1+ microglia also contained Aβ (**K**, merged). Scale bar in A = 40 µm (**A**–**C**), 65 µm (**D**–**K**).

To investigate the involvement of microglia in the spreading process, Iba-1 staining was performed. It was possible to distinguish two forms of microglia: round microglia (Figure 10A) and ramified microglia (Figure 10B,C). Quantitative analysis showed that the addition of the collagen hydrogel alone activated round Iba1+ microglia in all areas (Table 2). Compared to the collagen hydrogel load (as a control), the application of Aβ_42_ significantly increased the number of round and ramified microglia in area 1 (the area where the hydrogel was applied), and Iba1+ microglia were also activated in area 3 after application of Aβ_42_ (Table 2). The application of aggregated Aβ_42_ caused a more prominent activation of Iba1+ microglia in area 3 (Table 2). In order to study if microglia take up Aβ, co-localization experiments were performed with Iba1 (Alexa 488, green, Figure 10D,E), Aβ (A546, red, Figure 10F,G) and nuclear DAPI (blue) (Figure 10H–K). Quantitative analysis showed 59.6 ± 2.8% co-localization between Aβ and Iba1+ microglia (10 ± 1.2 microglia/field counted, *n* = 7).

### 3.10. Effects of Beta-Amyloid Spreading on Cholinergic Neurons

**Figure 11:**
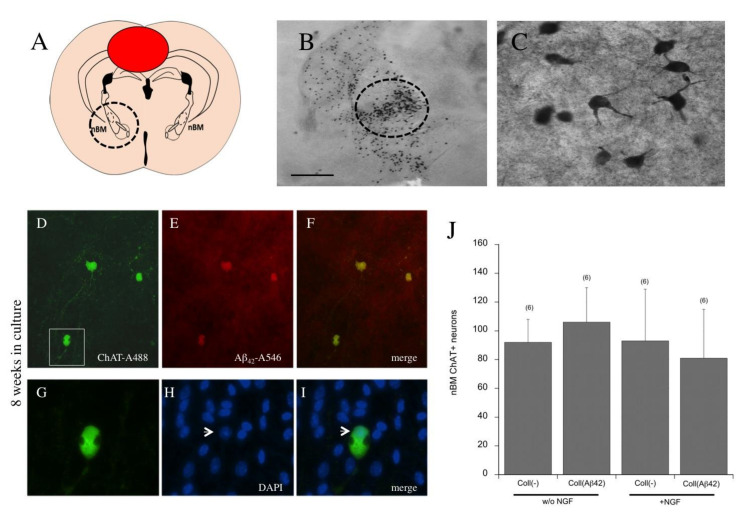
*Effects of beta-amyloid spreading on cholinergic neurons.* Brain slices of the basal nucleus of Meynert (nBM) were prepared and a collagen hydrogel loaded without or with human-beta-amyloid-42 was placed into the cortex (**A**, red) and after eight weeks of incubation with or without nerve growth factor (NGF) the cholinergic neurons were stained for choline acetyltransferase (ChAT) and counted. Cholinergic neurons were counted in the nBM (see circles in **A,B**) and showed a strong neuronal staining with nerve fibers (**C**). Co-localization experiments showed cytoplasmic staining of ChAT (**G**, green) in DAPI+ neurons (**H**, blue) in the merged picture (**I**). Co-localization showed that beta-amyloid (Alexa546, red, **E**) appeared as a diffuse staining around the cholinergic neurons (Alexa488, green, **D**), as shown in the merged picture (**F**). The white square (in (**D)**) shows a magnification of (**G**–**I**). Quantitative analysis shows that application of hAβ_42_ onto the brain slices did not markedly affect the survival of cholinergic neurons, neither when cultured with nor without (w/o) NGF (**J**). Statistical analysis was performed by one-way ANOVA and Fisher LSD post hoc test. Values are given as means ± SEM; values in parenthesis give the number of analyzed mice. Scale bar in B = 180 µm (**B**), 70 µm (**C**), 90 µm (**D**–**F**) and 25 µm (**G**–**I**).

To identify whether the spread of Aβ_42_ can damage cholinergic neurons, brain slices of the nBM were prepared and a collagen hydrogel with an Aβ_42_ load was placed at the cranial side of the slice (Figure 11A,B). In the nBM (Figure 11B, circle), several strong ChAT+ cholinergic neurons, showing distinct cytoplasmatic staining and arborization (Figure 11C), were visible after application of a collagen hydrogel with an Aβ_42_ load. The co-localization revealed the survival of cholinergic neurons with DAPI+ nuclei after eight weeks (Figure 11G–I) and a diffuse Aβ staining surrounding the cholinergic neurons (Figure 11D–F). Quantitative analysis highlighted that application of hAβ_42_ onto the brain slices did not markedly affect the survival of cholinergic neurons, regardless of whether they were cultured with or without nerve growth factor (NGF) (Figure 11J).

## 4. Discussion

In the present study, we demonstrated that human Aβ_42_ applied to the donor slice via a collagen hydrogel spreads from the donor slice over to the adjacent target slice. This spreading is accompanied by microglial activation and elimination of toxic hAβ_42_, which prevents cholinergic cell death.

### 4.1. Viability of Organotypic Brain Slices to Study Spreading

Organotypic brain slices have been extensively used to study spreading of Aβ and tau in AD or alpha-synuclein in Parkinson´s disease (see [37].). Organotypic brain slices have been well established in our laboratory for nearly 25 years [23]. Brain slices are three-dimensional networks of all brain cells that do not lose the cytoarchitectural structure of the brain when incubated for several weeks. As neuronal survival in organotypic brain slices is dependent on initial tissue thickness, organotypic brain slices are prepared from postnatal day 10 pups. Slices with a large thickness do not flatten during culturing, suggesting that the cells have low survival due to the reduced diffusion of media and oxygen as well as the insufficient waste removal. Therefore, we chose a slice thickness of 150 µm, which provides a good viability. In the present study we combined two coronal slices, one as the “donor slice” and the other as the “target slice”. Both slices were cut at the levels of the hippocampal formation, as this region is of predominant interest in memory formation. The“donor slice” served as the region where the Aβ peptides were loaded with the collagen hydrogel. The “target slice” was the adjacent slice where we wanted to observe the effects of spreading. As we could prepare many slices from one mouse pup, this model also reduced the number of animal experiments and contributed to the Three Rs. Although the slices have worked well in our hands, we fully characterized the viability in this study. We used propidiumiode as a marker of cell death and did not see any negative effects of application of collagen hydrogels alone or loaded with Aβ, compared to a positive control (H_2_O_2_). We also measured the release of lactate dehydrogenase into medium and also did not see any toxic effects after application of the hydrogels or Aβ, compared to a positive control (Triton). We confirmed viability with Western blot analysis using neuronal neurofilament, oligodendroglial MOG and microglial CD11b. There was some fragmentation of astroglial GFAP, as seen in previous studies, indicating some sort of activation of astrocytes in brain slices, and catalase was also partially increased. GFAP proteolysis/fragmentation is mediated by calpain at both C- and N-terminals, which results in a series of truncated GFAP breakdown products (38–44 kDa). It is not suggested that this GFAP fragmentation causes direct cell death, but is rather a consequence of the culturing as it is known that GFAP is increased at the borders of the slices. However, all these markers show a good viability of slices, and only in cases where slices did not flatten or were damaged, we did not include these slices in our experiments (approximately 20%).

### 4.2. Collagen Hydrogels as a Bolus to Apply Substances and Release

The major challenge in studying spreading is to apply substances locally in brain tissue at high concentrations. The application of compounds to slices has been extensively used by others, but faces the major problem that the amount of substance reaching the slice is not controllable. The compound of interest can be rapidly degraded in serum-containing medium: it may stick to the membranes or not enter the slice and become distributed all over the slice. Thus, to get a better controlled model, the direct application onto the slices was definitely better. Stereotaxic injection of low amounts (<1 µL) directly into the brain is a state-of-the-art procedure, but on slices it is more difficult. The application of 1–2 µL substances directly onto the slice causes rapid diffusion around many brain areas and is not local. Further there is a severe diffusion of the substance directly into the medium, again not controllable. Thus, in order to guarantee a local, stable and easy delivery of the substances of interest, we used the well-established collagen hydrogels for this “spreading study”.

Collagen and collagen hydrogels have been widely used to repair various tissues and structures, including those of the brain [38,39]. Collagen hydrogels loaded with growth factors such as NGF [32], GDNF [39] or FGF-2 [40] have been extensively used and characterized by us on organotypic brain slices. In these studies, we showed that substances can be loaded into collagen hydrogels and be released in a time-dependent pattern, and we showed that growth factor-loaded collagen hydrogels provided neuroprotection to neurons in organotypic brain slices [32]. We further showed that these collagen hydrogels were not toxic when applied onto brain slices and exhibited only a minor reactive gliosis [32]. In the present study, collagen hydrogels were used to load Aβ peptides. Collagen hydrogels have a size of approximately 2 mm and can be easily prepared, loaded, handled and placed onto the slices. In fact we showed that these collagen hydrogels are stable for at least 7–14 days and degrade over time. As the hydrogel is placed permanently on the brain slice and does not move, it is not likely that collagen transports the peptides along the slices or in the medium.

Our release data showed that only a minority of the loaded Aβ was released into the medium. It seems likely that (1) Aβ stuck to the slice and spread over; (2) Aβ was released and degraded in the medium; (3) Aβ was degraded in slices, e.g., by neprilysin; (4) Aβ was modified in the collagen and no longer detected by the antibody; or (5) Aβ spontaneously aggregated and could not be released any longer. Interestingly, the concentration of Aβ_40_ was lower in the medium, pointing to a different metabolism when applied to the slices. More experiments on the metabolism of Aβ in medium are necessary, especially to explain the effects on Aβ_40_. However, neither Aβ_40_ nor Aβ_42_ reached a maximal release into the medium and almost 50% was incorporated in the slices. Our release experiments only showed that Aβ could be released from the collagen hydrogel into the medium; however, it cannot be compared to either cerebrospinal fluid (CSF) or plasma. The human control levels of Aβ_42_ in CSF are approximately 800 pg/mL and in plasma approximately 60 pg/mL, while the levels of Aβ_40_ in CSF are approximately 10 ng/mL and in plasma approximately 200 pg/mL. Our data are clearly not comparable, as we did not have a blood–brain barrier between medium and slices, and also the application of exogenous Aβ was not directly comparable to a human situation. A limitation of the release data was that we only did the experiment once per time-point, but the release data were very stable and showed a clear time-dependency, thus giving a clear indication that collagen slowly released the peptides into the medium.

### 4.3. Only Human Beta-Amyloid_42_ Has Spreading Potential

In the present study we tested several Aβ peptides including respective controls. The rationale for using human Aβ was that, in contrast to murine Aβ, it tends to have a higher aggregation and a more pronounced deposition. In addition, murine and human Aβ differ in 3 amino acids at the residues 5, 10 and 13 [33,41]. Human Aβ_40_ and Aβ_42_ are the most common isoforms in humans generated by the amyloidogenic pathways, with human Aβ_40_ prevailing over human Aβ_42_ in a ratio of 9:1. Human Aβ_42_ aggregates nearly four times faster compared to human Aβ_40_. Also, human Aβ_42_ exerts neurotoxicity higher than human Aβ_40_ and the majority of plaques consist of Aβ_42_ species [41]. The human Aβ_42_ tends to aggregate into dimers and trimers and later into compact Aβ plaques [41]. In the present study we could produce stable and aggregated Aβ and confirmed the state of aggregation using Western blot analysis. The peptides were loaded into the hydrogels and we cannot exclude the possibility that they also aggregated in the hydrogel over time. We have no indication that collagen affected the process of aggregation.

Experiments with several controls were performed to show the specificity of the staining. Another protein that has been described to be abundant in plaques and to exert neurotoxic effects is pyrGlu-Aβ, which is formed by a glutaminyl-cyclase by adding glutamate to an N-terminal truncated Aβ, resulting in pyroglutamate-3 Aβ. The shorter Aβ_25-35_ peptide particularly contains the neurotoxic and hydrophobic properties that enable aggregation, and it has been identified as the active fragment of Aβ.

In the present study we used a commercial, well-characterized antibody (clone E610) and verified the specificity by Western blot analysis as well as in immunostainings. No staining was seen when controls (PBS or an empty collagen hydrogel) were applied. Nor was any staining observed after application of murine Aβ, pyrGlu-Aβ or Aβ_25-35_ and the application of pure Aβ_42_ (without collagen) also did not show any immunoreactive staining, suggesting that the application in the collagen hydrogels allowed a stable and slow release of Aβ. This was probably due to the rapid diffusion of liquid Aβ_42_ through the membrane into the medium, which is why the slow release of Aβ_42_ from collagen hydrogels was essential in this set of experiments. Only the application of reversed Aβ led to rather ambiguous results, as a slight positive staining appeared in some areas, an issue which must be verified in additional experiments. Using co-localization studies, we showed that the Aβ staining was intracellular, probably located in microglia (see Section 4.8). Aβ staining was stronger in the vicinity of the nuclei with bright fluorescence, which could indicate some forms of apoptosis and could suggest that Aβ uptake affects the viability of the cells.

### 4.4. Spreading in Transgenic APP Mice

The transgenic APP_SweDI AD model carries the Swedish, Dutch and Iowa mutations and begins to form insoluble Aβ in the brain after six months of age. As it is of transgenic nature it does not reflect the sporadic form of AD. As previously mentioned, AD is, in more than 99% of the cases, a non-genetic disease, which appears in humans aged > 60 years. Thus, transgenic AD models never represent the sporadic form of AD (see our review [21]). In the present study we used transgenic mice at the age of 10 days to test whether application of human Aβ_42_ could potentiate any Aβ pathology. As mentioned earlier, the APP_SweDI transgenic mice show a plaque pathology at an age > 6 months, and we expected to induce plaques in our slice model. In fact, in the present study we were able to show that application of human Aβ_42_ in collagen hydrogels potentiated the Aβ depositions in the donor and target slices. At this stage only some forms of larger depositions were seen in area 3 (donor slice). However, in area 5 in the target slices a reduced Aβ staining was seen, indicating an enhanced metabolism and eventually higher Aβ degrading activity or pronounced microglial activity in the slices taken from transgenic mice. However, at no stage did we find distinct, large extracellular-typical plaques similar to those in the transgenic mouse brain.

### 4.5. The Role of Different Brain Areas in Spreading

The “spreading hypothesis” suggests that yet unknown events activate a pathological protein (similar to prions) that originates in specific brain areas and spreads over the whole brain [5,42]. The Aβ pathology begins in the neocortex and then involves the hippocampus, the striatum, basal forebrain, diencephalic nuclei and finally the brain stem (see [16]). In contrast, tau aggregates are first found in the locus coeruleus and entorhinal cortex before progressing to the hippocampus and cortex [43]. Alpha-synuclein is suggested to be absorbed by the stomach/duodenum and transported via the vagal nerve to the brainstem, from where it spreads into the brain [44]. Organotypic brain slices are an efficient tool to study the spreading starting from the origin in the “donor cortex”. In order to study if spreading of human Aβ_42_ is influenced by different brain areas, we connected the target slices to (a) the hippocampus as the control, (b) the dorsal striatum slice, (c) the ventral striatum slice including the limbic areas or (d) the brain stem. Our results showed that the cerebral cortex and the hippocampus displayed depositions of Aβ after application of the collagen hydrogel after eight weeks of incubation. These results confirmed that the hippocampus is an area affected early by the spreading of aggregated Aβ. We were also able achieve a significant, but nevertheless only slight, spreading of the monomeric Aβ_42_ to some regions of the adjacent “target slice”. When Aβ_42_ was applied to the dorsal striatum, more Aβ_42_ was found in area 1 (collagen hydrogel application area) and area 5 (target), suggesting a slower release from the collagen hydrogel and greater propagation to the target regions. After application of Aβ_42_ to the ventral (limbic) striatum, less Aβ_42_ was found in area 1 (collagen hydrogel) and a markedly reduced amount in areas 6 and 7 in the target regions. When Aβ_42_ was applied to the brain stem, less Aβ_42_ was found in the area 1, but in contrast to the ventral striatum, more Aβ_42_ spreading was seen in the target area. Three different mechanisms could be described: (1) an increase in the collagen hydrogel followed by an increase in the target area caused by the dorsal striatum and a decrease in the collagen hydrogel followed by either (2) a decrease in the target area caused by the ventral striatum or (3) an increase in the target area caused by the brain stem. These mechanisms and pathways for this differential spreading influenced by the brain areas are not fully understood and need to be further studied in future work.

### 4.6. Effects on Murine Endogenous APP

Next, we wanted to explore if the application of exogenous Aβ_42_ affects the expression of endogenous murine APP. In order to evaluate this, we used murine specific qRT-PCR and showed that none of the treatments with human Aβ_42_ affected the expression of murine APP. This clearly shows that exogenously applied human Aβ_42_ does not interact with the endogenous murine APP/Aβ and that all effects observed on spreading were clearly caused by the application of the human exogenous Aβ_42_. In this context it would be interesting to also measure endogenous BACE-1 and γ-secretase activities after application of the Aβ peptides.

### 4.7. Astroglial Responses

GFAP is a marker for reactive astrocytes in the brain and becomes activated in AD, especially when Aβ plaques are surrounded by reactive astrocytes. Our data showed that the collagen hydrogel slightly activated reactive astrocytes at the edges and in the hydrogel. This was clearly a reaction of the application of the collagen hydrogel onto the tissue, which confirms previous results in our slice model. In addition, our data showed that the exposure to Aβ-loaded hydrogel (either monomeric or aggregated) further caused an increase in GFAP expression. This effect was also time-dependent and increased significantly within eight weeks of cultivation. In the hippocampus, the GFAP reaction was only moderate when the empty hydrogel was placed but increased after loading of monomeric Aβ. Interestingly, the GFAP reactivity in the hippocampus dramatically increased after loading of aggregated Aβ. This clearly shows that the toxicity of aggregated Aβ is higher and also that the hippocampus is more sensitive to Aβ exposure. In contrast to the direct effects of the collagen hydrogel, we did not find that the spreading of Aβ in other brain areas caused a marked GFAP reactivity. What this clearly shows is that the reactive astrogliosis is caused by the mode of application and that it does not occur as a consequence of the spreading of Aβ. Furthermore, we did not see the typical plaque pathology surrounded by GFAP+ astrocytes. Finally, we also wanted to investigate if astrocytes can phagocyte Aβ. In fact, there is weak evidence that astrocytes can target plaques [7] and directly attach to them and contribute to plaque elimination. However, the process of how astroglia can eliminate plaques is still unclear. Our data provide evidence that the astrocytes activated in the slices did not phagocyte human Aβ_42_.

### 4.8. Microglial Responses

Neuroinflammation is another severe and prominent pathology during AD progression that leads to the activation of microglia. To date, it is not clear if neuroinflammation in AD is a primary event or the cause of the excessive plaque depositions. Indeed, microglia get activated and migrate to the plaques in order to phagocyte and eliminate plaques. Microglia are a double-edged cell [45,46,47,48,49] that, on the one hand, can protect and repair damaged neurons and release protective growth factors and, on the other hand, when microglia recognize that a neuron is degenerating, produce pro-inflammatory cytokines and eliminate the cell. In the case of AD, microglia get activated during the early stage of plaque depositions and they phagocyte and eliminate plaques. However, if the severity of the disease and the number of plaques increases, microglial activity becomes impaired and phagocytic activity diminishes [45,46,47,48,49].

Microglia are complex cells that can be round or amoeboid and migrate to lesion sites. They can also be present in the brain in a resting form, get activated and ramified, express many markers of activation and, in a late stage, transform into macrophages [45,46,47,48,49]. In the present study we used the well-known Iba1 antibody to detect microglia and we were able to see round as well as ramified forms. Our data showed that microglia already become activated due to the application of the collagen hydrogel. This is not really unusual as the application of an exogenous material (collagen) causes microglial activation. However, when we compared the effects of the collagen hydrogel loaded with Aβ versus the empty collagen hydrogel, we also found a clear pronounced activation of microglia caused by Aβ application. This effect was most prominent in areas 1 and 3. Our co-localization data showed that activated microglia indeed also stained positive for Aβ and we concluded that the spread Aβ had been taken up by microglia. This was indeed in line with the hypothesis that microglia phagocyte human Aβ_42_ and aim to eliminate them. Hellwig et al. [50] treated cultured hippocampal slices from neonatal mice with Aβ_42_ and only when slices were treated with clodronate to eliminate microglia did these slices develop Thioflavin-S-positive plaques after two weeks. Activated microglia were found in the halo of Aβ plaques, indicating an interaction with Aβ_42_ oligomers and fibrils. In fact, there is clear evidence that activated microglia phagocyte Aβ in AD [46,49,50,51,52,53] and our data are in full agreement and confirm that Aβ is taken up by microglial cells. Interestingly, the microglial activity is reduced during late stage AD progression and it is considered that this reduced activity is directly linked with the rapid progression of the Aβ_42_ plaques [54]. In fact, already in 1999 Chung et al. [51] showed that microglia take up Aβ; however, the majority of the Aβ was not degraded but released. It seems possible that CD33 plays a role in this process, as CD33 inhibited the uptake and clearance of Aβ in microglial cell cultures [55]. This all may explain why we saw such a high Aβ load in the microglia in our brain slice cultures. Our co-localization studies showed intracellular staining which was probably microglia. As discussed, hAβ_42_ staining was somewhat stronger in the vicinity of the nuclei with bright fluorescence and it could be that the uptake of Aβ into microglia causes their apoptotic cell death. This would be in line with findings that suggest that phagocytic cells undergo apoptosis in order to eliminate toxic plaques. This could be the reason why microglial function is diminished in late AD. Nevertheless, more work is necessary to trace the route of the Aβ load or to selectively kill microglia and follow up Aβ plaque production in our slices. In addition, it is worth noting that a similar mechanism may exist for tau, as microglia also take up tau and release it but are incapable of neutralizing its seeding activity [56]. Our data show that the majority of microglia (50%) contributed to the uptake and elimination of Aβ from the brain tissue. The phagocytosis of Aβ by microglia may have prevented the formation of plaques, as we have not yet seen any plaque deposition in the brain slices. At this point, it would be interesting to eliminate microglia (e.g., with clodronate) and culture brain slices for a longer time period to see whether plaques develop or not.

### 4.9. Effects of Spreading on Cholinergic Neurons

In AD, cholinergic neurons degenerate and a loss of the neurotransmitter acetylcholine directly correlates with cognitive decline. The “selective vulnerability hypothesis” suggests that specific neurons are more vulnerable and more selectively as well as more easily “infected”, such as, e.g., the cholinergic neurons in AD. Thus, in the present study we wanted to see if the spreading of human Aβ_42_ had any toxic effects on cholinergic neurons. In our research group, we have extensive experience with the cultivation of cholinergic neurons of the nBM [22,23,30] and we showed that cholinergic neurons in chopper slices are highly dependent on the addition of NGF, while in coronal brain slices this dependence is smaller. In order to study the effects of Aβ_42_, we used the well-established coronal slice model and prepared sections of the nBM and incubated them with or without NGF for eight weeks. We were able to show that these cholinergic neurons indeed survived well when incubated for eight weeks. While in the previous model we cultured two half-brain slices (donor and target), in this experiment we used whole coronal slices and applied the collagen hydrogel loaded with Aβ_42_ directly on top of the hippocampus. Our data showed that neither the application of Aβ_42_ nor the withdrawal of NGF affected the survival of cholinergic neurons. We concluded that the toxic Aβ_42_ spread over to the nBM, but that Aβ_42_ was captured and eliminated by activated microglia. In fact, around the nBM we mainly saw a diffuse Aβ_42_ staining and less Aβ_42_ incorporated by microglia, indicating that the microglia protect against Aβ_42_ toxicity. This effect was seen when slices were incubated both with and without NGF, implying that the surrounding cells, e.g., activated astrocytes or microglia, can contribute to the survival of the cholinergic nBM neurons. Indeed, this model of the nBM is definitive evidence that application of toxic Aβ_42_ does not necessarily cause cholinergic cell death in our spreading model. Supporting evidence in the literature suggests that microglia take up Aβ and thus reduce the Aβ load in the brain, which over the long term contributes to neuroprotection in AD [49,52,57]. Richter et al. [52] recently proved the neuroprotective role of microglia cells against Aβ toxicity in organotypic hippocampal slice cultures. Our data are in full line with this finding and show for the first time that elimination of hAβ_42_ protects cholinergic nBM neurons, although it would be worth also investigating the microglial activation and elimination of Aβ in slices of the nBM in further studies. Alternatively, we cannot exclude the possibility that in our model the number of activated microglia was increased, which would explain (at least partly) the uptake of Aβ.

### 4.10. Spreading Hypothesis

The “prion hypothesis” is based on the idea that neurodegenerative diseases are caused by a systemic aggregation and transport of misfolded proteins in the brain through axonal pathways [9,58], suggesting that misfolded Aβ can recruit and corrupt healthy Aβ peptides in the brain, causing the formation of aggregated Aβ. Studies on mice have shown that different injection sites of aggregated Aβ led to a varying distribution of the misfolded peptide itself [59,60,61]. The “spatiotemporal spreading hypothesis” implies a radial propagation of the predominantly extracellular misfolded Aβ from one hotspot to another spatially proximal region in a radial manner [59]. This theory coincides with the properties of APP as a transmembrane protein and the fact that Aβ originates as a cleavage product of the extracellular domain [59]. The “transsynaptic spreading hypothesis” puts focus on the dense interconnection of neuronal networks, such as, for example, the entorhinal cortex and the hippocampal formation [62]. However, research on the transsynaptic spreading hypothesis has led to unsatisfactory results, as the results of Aβ spreading were not as explicit, as for tau and alpha-synuclein and spreading models based on the hypothesis failed to reproduce the spatiotemporal Aβ spreading in vivo [59]. Therefore it could be that both the spatiotemporal and transsynaptic spreading occur simultaneously or at least successively, with the latter preceding the former [63]. Our results showed that the aggregated and monomeric Aβ_42_ spread to areas which were spatially near or fibrillary connected to the inoculation site of the hydrogel. Starting from the inoculation site, both the aggregated and the monomeric Aβ_42_ spread further into the cerebral cortex, which is fibrillary connected to the inoculation site, and into the hippocampus, which has no fibrillar connection to the inoculation size but is spatially close. Additionally, the aggregated Aβ_42_ form showed greater and more prominent spreading, which was more specific to the hippocampus than the monomeric form. This hypothesis of the occurrence of both the extracellular and the intracellular spreading has also been proposed for tau, which is likewise assumed to propagate in a prion-like fashion [64].

### 4.11. Mechanism of Spreading

The mechanism of spreading of the Aβ pathology is not clear, but four hypotheses can be considered [9,16,65]. (1) First, it is suggested that Aβ spreading is dependent on axonal connections and that Aβ peptides are transported anterogradely in the neuron to the synapse, where they are released. This mode of actions needs active neuronal uptake of Aβ but so far there is no conclusive evidence for active transport of Aβ along the neurons [66]. Different modes of actions have been discussed: via secreted small vesicles, the exosomes, or via thin membranous tunnel nanotubes connecting neurons for active or “non-classical” uptake of the naked “infectious” protein. Alternatively, retrograde transport in neurons may also be possible. In our brain slice model, most long axonal connections were cut due to the dissection of the brain slices; thus, we think that this mode of action cannot occur in our brain slice model. Additionally, we have no evidence of neuronal uptake. (2) Second, there is more evidence that the Aβ peptides just simply diffuse in the extracellular space over long distances. Indeed, diffusion of peptides over long distances has been recognized and is known as volume transmission [67]. Our data more likely support this mode of spreading, and we think that the Aβ peptides diffuse in the brain slices. However, we cannot exclude the possibility that Aβ peptides are also released into the medium and taken up again in other brain areas in the slices; however, this seems unlikely, as we do not see a homogenous distribution. (3) Third, it is possible that the spreading arises *de novo* in different regions depending on alternative regional production and degradation in a “prion-like” pattern. This is not supported by our data, as we used wildtype murine slices and applied exogenous human Aβ_42_. (4) Finally, it is possible that migrating cells transport the Aβ peptides in the brain. In fact, round amoeboid microglia are the most potent migrating cells and rapidly become activated, differentiate and migrate to lesion sites in the brain. Although we do not have experimental evidence from live cell imaging, we can see that mainly round amoeboid microglia take up Aβ and they are also found all over the brain slices.

### 4.12. Translation to Human AD

The present study definitely provides proof-of-principle that human Aβ_42_ can spread over on mouse brain tissue and can be eliminated by activated microglia. The question now arises how these data can be translated to humans. (1) First of all, there are already clear indications that, in human AD, microglia are activated and become dysfunctional in late AD. (2) One always asks how mouse and human can be compared. As we do not have any other possibilities to investigate spreading in vivo, mouse models are a compromise and can show proof-of-principle on brain tissue. (3) AD is a disease of the aging brain and sporadic AD needs 20–30 years to develop, so one may clearly dispute that culturing of brain slices for eight weeks may have any pathological relevance for human AD. Unfortunately, we are limited in our culture models and culturing of adult slices is very complex and tricky. (4) Brain slices were taken from postnatal day 10 mice and clearly cannot be directly compared to adult human brains. Again, this was a compromise and was clearly better than using isolated embryonic neurons or malignant cells. (5) Generally, in our society, experiments with living animals should be reduced, thus also limiting some aspects of data interpretation, and again our slice model contributes to the Three Rs and reduces animal pain. (6) Our data show that we did not see any typical large extracellular plaques, similar to those found in human AD. This could have been because the microglia prevent plaque deposition or just because the time of incubation was too short. It would be interesting to culture brain slices with Aβ loads for 10–12 months. (7) Our data also show that addition of hAβ42 alone did not cause the typical plaques; as stated by us and also others, it is very likely that other co-factors (such as, e.g., tau, apolipoprotein E4, metals, silent strokes or inflammation) may be necessary to induce plaques [33]. Using our brain slice model we are in a good position to test any single exogenous factor, which is not possible in vivo or in humans. (8) There is clear evidence in the literature that oligomeric Aβ is the toxic form; our data did not show that application of monomeric/dimeric or aggregated Aβ are directly toxic to cholinergic neurons; thus, clearly counteracting mechanisms must be considered, which are also present in the human brain. (9) In our model we used collagen only to apply the peptides locally; it is not suggested that collagen may play any role in the human brain or in AD. However, local application of collagen-loaded drugs may become a potent therapeutic target. We are just underway in showing that intranasal application of (non-toxic) collagen-loaded neprilysin eliminates plaques in transgenic mice [68]. (10) Finally our data cannot explain from where the toxic hAβ42 arises and how the pathology is initiated and progresses; this issue will be one of the most important questions in AD research in the future.

## 5. Conclusions

In summary, in the present study we showed that hAβ_42_ spreads over into a target slice when applied onto a donor slice. This spreading is accompanied by microglial activation and elimination of toxic hAβ_42_ which prevents cholinergic cell death. This study adds another piece of evidence to support findings that toxic hAβ_42_ is eliminated and trapped by microglia. Although the hAβ cascade hypothesis is still valid, it is becoming clear that hAβ alone does not account for AD pathologies; however, it is still essential to find therapeutic targets to counteract spreading and toxicity of hAβ_42_ in humans.

## Figures and Tables

**Table 1:** Quantification of spreading in organotypic co-slices.

Treatment	Area 1	Area 2	Area 3	Area 4	Area 5	Area 6	Area 7
PBS	0 ± 0 (6) ns	0 ± 0 (6) ns	0 ± 0 (6) ns	0 ± 0 (6) ns	0 ± 0 (6) ns	0 ± 0 (6) ns	0 ± 0 (6) ns
CollH (–)	0 ± 0 (20) vs	0 ± 0 (20) vs	0 ± 0 (20) vs	0 ± 0 (19) vs	0 ± 0 (20) vs	0 ± 0 (20) vs	0 ± 0 (21) vs
CollH (hAβ40)	0.4 ± 0.4 (5) ns	0.4 ± 0.4 (5) ns	0 ± 0 (5) ns	0 ± 0 (5) ns	0 ± 0 (5) ns	0 ± 0 (5) ns	0 ± 0 (5) ns
CollH (hAβ42)	54.7 ± 9.7 (24) ***	26.2 ± 7.3 (24) ***	9.8 ± 2.4 (24) ***	3.8 ± 1 (24) **	1.6 ± 0.5 (22) *	5.5 ± 1.6 (22) *	2.1 ± 0.8 (24) **
CollH (AGG hAβ42)	60 ± 8.2 (18) ***	32.3 ± 9.5 (18) **	7.7 ± 1.7 (17) **	4.8 ± 1.1 (18) ns	1.3 ± 0.6 (19) *	9.2 ± 2.6 (19) ***	1.6 ± 0.4 (19) ns
hAβ42 (w/o CollH)	0.3 ± 0.3 (6) ns	4.7 ± 4.7 (6) ns	0 ± 0 (6) ns	0 ± 0 (6) ns	0 ± 0 (6) ns	0.2 ± 0.2 (6) ns	0 ± 0 (3) ns
CollH (pyrG-Aβ)	1 ± 1 (3) ns	9.3 ± 6.4 (3) ns	0 ± 0 (3) ns	0 ± 0 (3) ns	0 ± 0 (3) ns	1.3 ± 0.7 (3) ns	0 ± 0 (3) ns
CollH (Aβ25-35)	0 ± 0 (3) ns	0 ± 0 (3) ns	0 ± 0 (3) ns	0 ± 0 (3) ns	0 ± 0 (3) ns	0 ± 0 (3) ns	0 ± 0 (3) ns
CollH (hAβ42Rev)	14.7 ± 5.0 (6) ns	13.8 ± 3.5 (6) ns	16.6 ± 1.6 (6) ***	16.7 ± 1.0 (6) ***	0.5 ± 0.2 (6) ns	1.0 ± 0.4 (6) ns	0 ± 0 (6) ns
CollH (mAβ42)	0 ± 0 (6) ns	0 ± 0 (6) ns	0 ± 0 (6) ns	0 ± 0 (6) ns	0 ± 0 (6) ns	0 ± 0 (6) ns	0 ± 0 (6) ns

Organotypic co-slices were prepared and after one week in culture a bolus was applied on the donor slice and slices then incubated for eight weeks and quantified in seven areas: area 1 (location of the hydrogel), areas 2 and 3 (donor slice), area 4 (borders) and areas 5–7 (target slice). Slices were fixed and stained for beta-amyloid and then quantified using computer-assisted imaging (pixels 400–8000). Values are given as means ± SEM; values in parenthesis give the number of analyzed animals. Statistical analysis was performed using ANOVA with a Fisher LSD post hoc test, where *p* < 0.05 represents significance compared versus (vs) CollH(-) (* *p* <0.05; ** *p* < 0.01, *** *p* < 0.001; ns, not significant). Values 1–7 indicate the seven different areas, as indicated in Figure 1B. Abbreviations: PBS, phosphate buffered saline; CollH, collagen hydrogel; Aβ_42_, beta-amyloid_42_; AGG, aggregated; pyrG, pyroglutamate; w/o, without.

**Table 2:** Iba1 positive microglia in organotypic brain slices after beta-amyloid spreading.

Group		Area 1	Area 3	Area 5	Area 7
PBS	Round	9.4 ± 1.7 (6) **	7.4 ± 0.5 (6) **	4.6 ± 0.5 (6) ***	3.6 ± 0.6 (6) ***
	Ramified	1.6 ± 0.3 (6) ns	2.3 ± 0.5 (6) ns	1.4 ± 0.3 (6) ns	0.2 ± 0.2 (6) **
	Total	11 ± 2.0 (6) **	9.7 ± 1.0 (6) ns	6.0 ± 0.8 (6) ns	3.8 ± 0.8 (6) ns
CollH(-)	Round	16.2 ± 0.9 (6) vs	17.6 ± 0.6 (6) vs	14.6 ± 1.4 (6) vs	12.8 ± 1.2 (6) vs
	Ramified	4.1 ± 1.4 (6) vs	2.3 ± 0.8 (6) vs	3.5 ± 0.9 (6) vs	3 ± 0.4 (6) vs
	Total	20.3 ± 2.3 (6) vs	19.9 ± 1.4 (6) vs	18.1 ± 2.7 (6) vs	15.8 ± 1.6 (6) vs
CollH(Aβ42)	Round	25.3 ± 1.1 (6) ***	24.6 ± 1.2 (6) ***	15.4 ± 0.9 (6) ns	12.6 ± 2.3 (6) ns
	Ramified	10.2 ± 1.3 (6) ***	5.8 ± 1.2 (6) ns	4.2 ± 1.2 (6) ns	3.6 ± 1.5 (6) ns
	Total	35.5 ± 2.4 (6) **	30.4 ± 2.4 (6) ns	19.6 ± 2.1 (6) ns	16.2 ± 3.8 (6) ns
CollH(AGG Aβ42)	Round	22.8 ± 1.1 (6) ***	25.6 ± 1.8 (6) ***	16 ± 1.4 (6) ns	13.4 ± 1.8 (6) ns
	Ramified	8.5 ± 0.9 (6) ns	12.4 ± 3.1 (6) ***	1.5 ± 0.6 (6) ns	2.4 ± 0.5 (6) ns
	Total	31.3 ± 2 (6) **	38 ± 4.9. (6) **	17.5 ± 2 (6) ns	15.8 ± 2.3 (6) ns

Organotypic co-slices were prepared and after one week in culture, collagen hydrogels were loaded with human beta-amyloid(42), and applied on the donor slice and slices were then incubated for eight weeks. Slices were fixed and stained for Iba1 and then quantified by counting round and ramified cells at 20×. Values are given as means ± SEM; values in parenthesis give the number of analyzed animals. Statistical analysis was performed using ANOVA with a Fisher LSD post hoc test, where *p* < 0.05 represents significance versus (vs) a control CollH(-) (** *p* < 0.01, *** *p* < 0.001; ns not significant). Abbreviations: PBS, phosphate buffered saline; CollH, collagen hydrogel; Aβ_42_, human beta-amyloid 42; AGG, aggregated. For the areas, we refer to Figure 1B.

## Data Availability

Not applicable.
